# Preventing surgical complications: A survey on surgeons' perception of intra-articular malleolar screw misplacement in a cadaveric study

**DOI:** 10.1186/1754-9493-5-24

**Published:** 2011-10-04

**Authors:** Vincenzo Giordano, Arthur FS Gomes, Ney P Amaral, Rodrigo P Albuquerque, Robinson ES Pires

**Affiliations:** 1 Serviço de Ortopedia e Traumatologia Prof. Nova Monteiro, Hospital Municipal Miguel Couto, Rio de Janeiro, RJ, Brasil; 2 Serviço de Ortopedia e Traumatologia, Hospital Felício Rocho, Belo Horizonte, MG, Brasil

**Keywords:** Ankle, Fracture, Cadaver, STARD

## Abstract

**Background:**

Intra-articular hardware penetration can occur during osteosynthesis of ankle fractures, jeopardizing patients' outcomes. The intraoperative recognition of misplaced screws may be difficult due to the challenge of adequate interpretation of specific radiographic views. The present study was designed to investigate the diagnostic accuracy of standardized radiographic ankle views to determine the accuracy of diagnosis for intra-articular hardware placement of medial malleolar screws in a cadaveric model.

**Methods:**

Nine preserved human cadaveric lower extremity specimens were used. Under direct visualization, two 4.0 mm cancellous screws were inserted into the medial malleolus. Each specimen was analyzed radiographically using antero-posterior (AP) and mortise views. The X-rays were randomly uploaded on a CD-ROM and included in a survey submitted to ten selected orthopaedic surgeons. The "Standards for Reporting of Diagnostic Accuracy" (STARD) questionnaire was used to determine the surgeons' perception of accuracy of screw placement in the medial malleolus. The selection of items was based on evidence whenever possible, therefore the "inconclusive" category was added. Inter and intraobserver variations were analyzed by kappa statistics to measure the amount of agreement.

**Results:**

There was a poor level of agreement (kappa 0.4) both in the AP and in the mortise view among all the examiners. Associating the two x-rays, the agreement remained poor (kappa 0.4). In the cases in which there was a diagnosis of articular penetration, there was a poor agreement related to which of the screws was intra-articular. The number of "inconclusive" responses was low and constant, without a statistically significant difference between the subspecialists

**Conclusion:**

The routine intraoperative radiographic imaging of the ankle is difficult to interpret and unreliable for detection of intra-articular hardware penetration. We therefore recommend to reposition medial malleolar screws intraoperatively if there is any doubt regarding inadequate screw placement.

## Introduction

Transverse fractures of the medial malleolus present in several different patterns affecting all ages. The importance of anatomic reduction and rigid internal fixation in displaced fractures of the medial malleolus has been emphasized in order to achieve rapid return of normal function and to reduce the incidence of complications related to this fracture [1]. Stable fixation can be accomplished either by lag screws or K-wires with or without cerclage wiring [2-6]. The most important advantage of the leg-screw technique of osteosynthesis relies on the static interfragmentary compression, preventing the fracture site from gapping and rotating [2].

However, intra-articular hardware penetration and cartilage damage are potential disadvantages related to lag screwing, therefore reducing the chances of successful outcome [1]. In this scenario, rigorous intraoperative radiographic evaluation is mandatory to assess the position of the lag screws into the distal tibia fragment.

It is generally agreed that three standard views (antero-posterior (AP), mortise, and lateral) should be used routinely in the preoperative evaluation of ankle acute traumas [7-11]. However, the value and necessity of these incidences have been poorly investigated in the intraoperative situation of ankle fracture reduction and fixation [12-14]. We hypothesized that intraoperative recognition of misplaced screws may be difficult due to the challenge of adequate interpretation of specific radiographic views.

The present study was designed to investigate the diagnostic accuracy of standardized radiographic ankle views to determine the accuracy of diagnosis for intra-articular hardware placement of medial malleolar screws in a cadaveric model.

## Methods

Nine preserved human cadaveric lower extremity specimens were obtained from the Department of Legal Medicine (DLM) of our institution. This was the amount of the existing specimens in the DLM at the moment of the investigation. There was no gross evidence of deformities, previous injuries, or surgeries involving the lower extremities of these cadavers. There were seven male specimens and the mean estimated age was 67 years old.

**Figure 1:**
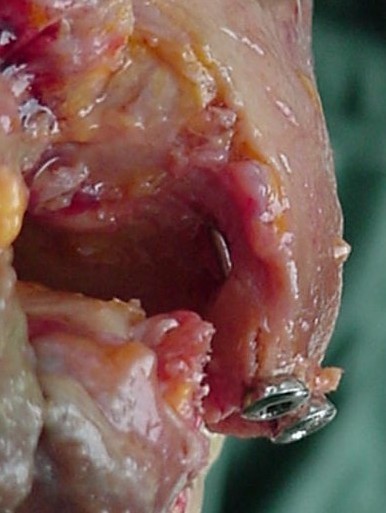
**Medial aspect of the ankle of one specimen showing the intra-articular penetration of the most anterior screw**.

**Figure 2:**
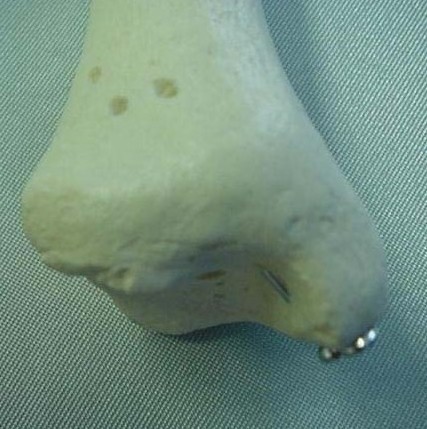
**Representation of the same situation on the plastic bone**.

**Figure 3:**
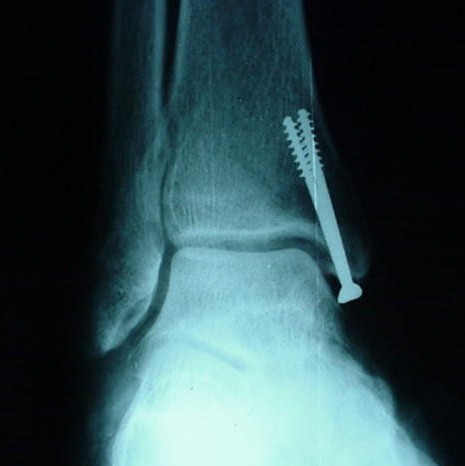
**Mortise view of the ankle of one specimen with the medial screws inserted**.

**Figure 4:**
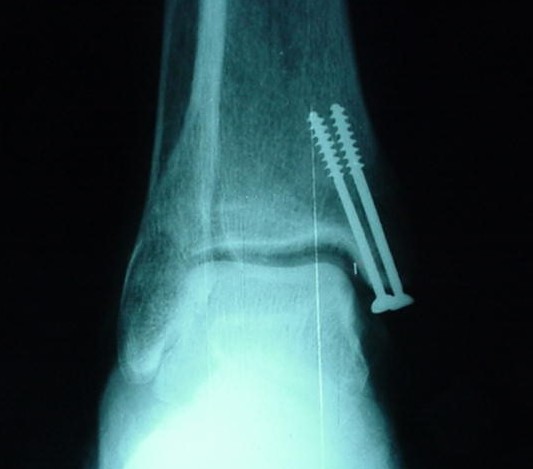
**Antero-posterior view of the ankle of the same specimen shown on Figure 3**.

Each specimen had a medial exposure of the ankle joint performed by the same author. The region was left intact, thus simulating a situation of anatomic reduction of a medial malleolar fracture. The medial malleolus was identified and completely detached from its soft-tissue insertions. This allowed the talus to be laterally dislocated, exposing the roof of the ankle joint. Under direct visualization, two 4.0 mm cancellous screws available in the AO (*Arbeitsgemeinschaft für Osteosynthesefragen*) small fragments set (*Synthes Brasil*, Brasil) were inserted into the medial malleolus. Four specimens had both lag screws placed extra-articularly, three had one lag screw placed intra-articularly, and two had both lag screws placed intra-articularly. All intra-articular screws penetrated the lamina splendens layer of the articular cartilage (approximately 50% of its diameter penetrated the joint itself) (Figures 1 and 2). The talus was then repositioned and the specimens were assessed radiographically with plain films (*Aktiengesellschaft Wittelsbacherplatz 2 D80333*™, *Siemens*, Germany), using standard technique (distance of 1 m, 4 mA/seg, 46 KV) (Figures 3 and 4).

**Figure 5:**
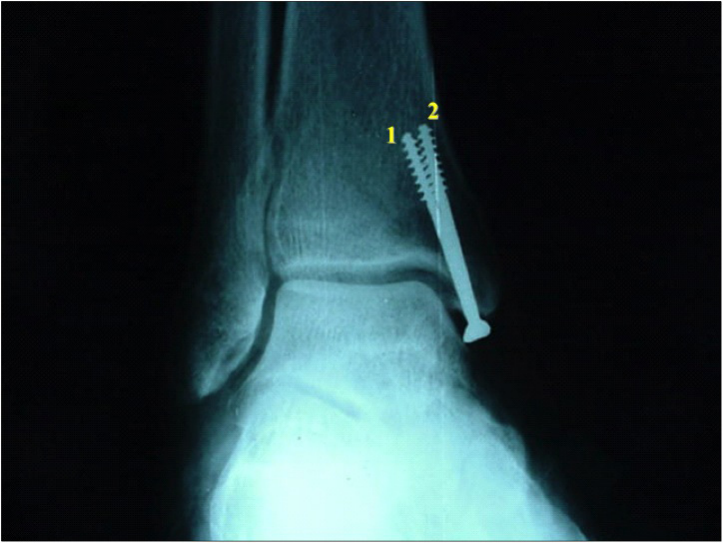
**Mortise view of the ankle of the same specimen shown on Figure 3 with the screw numbers as submitted in the questionnaire**.

**Figure 6:**
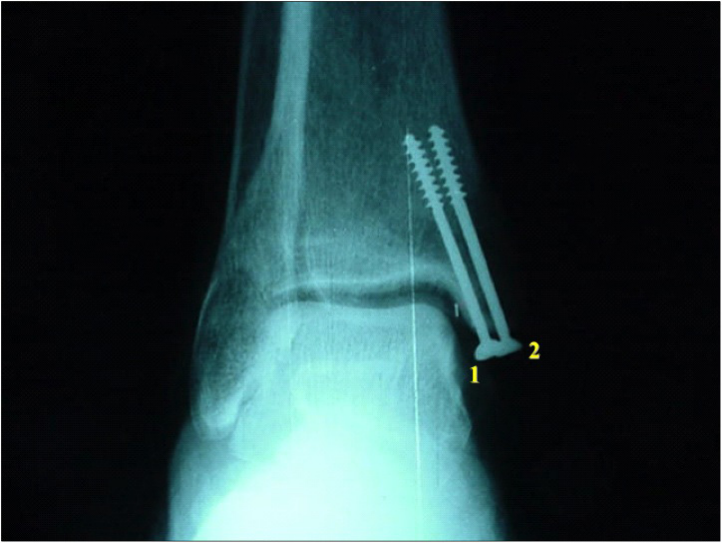
**Antero-posterior view of the ankle of the same specimen shown on Figure 4**. The observers were asked to refer screw positioning related to screws number 1 and 2.

Each specimen was studied on AP and mortise views. As the medial articular surface cannot be adequately demonstrated with the ankle lateral view, this incidence was judged to be unnecessary for the current investigation. The mortise view was made with the leg resting in a custom-made platform with 20 degrees of internal rotation. The films were photographed (*Cybershot*, *Sony*, Japan), randomly included in CD-ROMs, and viewed independently by ten orthopaedic surgeons: five Trauma Surgeons and five Foot and Ankle Surgeons (Figures 5 and 6). This approach aims at describing higher order agreement by assessing agreement among groups of observers of decreasing size in a stepwise manner.

Each physician was asked to answer a questionnaire over a 24-hour period and return it with the CD-ROMs (Table 1). The images were mixed in a random fashion and re-evaluated independently by the same observers with 2 weeks apart. All the interpreters had total freedom for enlarge the image, if one judged necessary to do that.

**Table 1:** Questionnaire applied to all the participants

Questions	Answers
1. Which view?	( ) AP ( ) Mortise ( ) Inconclusive
2. Do you think there is intra-articular hardware penetration?	( ) Yes ( ) No ( ) Inconclusive
3. If yes, which one (or both)?	( ) 1 ( ) 2 ( ) 1 and 2 ( ) Inconclusive

The questionnaire was elaborated following the Standards for Reporting of Diagnostic Accuracy (STARD) [15]. The selection of items was based on evidence whenever possible, therefore the "inconclusive" category was added. Inter and intraobserver variations were analyzed by kappa statistics, according to Svanholm et al and Brage et al [16,17]. The kappa-statistic was used to analyse agreement between more than two observers on an ordinal scale, on the basis of the analysis of every pair of observers [18]. The kappa-statistic measure of agreement was scaled to be one when there was perfect agreement and zero when the amount of agreement was what would be expected to be observed by chance. Intermediate values were defined as evidenced in Table 2.

**Table 2:** Statistical analysis (kappa-statistic measure of agreement)

Score	Level of agreement
• Zero	no agreement
• Below 0.5	poor agreement
• Between 0.75 and 0.5	good agreement
• Greater than 0.75	excellent agreement
• One	perfect agreement

## Results

### Intraobserver variation

Radiographic view - there was good agreement between the five Trauma Surgeons. The number of "inconclusive" responses was low and constant during the two readings. There was good agreement (kappa 0.8) among the five Foot and Ankle Surgeons, with values that were discretely lower than those of the Trauma Surgeons, without statistical significance (p > 0.05, ANOVA test) [18]. The number of "inconclusive" responses was low and constant during the two readings.

Intra-articular screw penetration - there was poor agreement (kappa 0.4) among Trauma Surgeons and Foot and Ankle Surgeons when the radiographic views were independently evaluated. Both subspecialists had difficulty in the diagnosis of joint penetration and in the definition of which screw was intra-articular.

The number of "inconclusive" responses was significantly lower between the Trauma Surgeons (p < 0.05) [18].

### Interobserver variation

Radiographic view - there was good agreement (kappa 0.8) among all of the examiners. The number of "inconclusive" responses was slightly lower among the Trauma Surgeons, without statistical representation (p > 0.05) [18].

Intra-articular screw penetration - there was a poor level of agreement (kappa 0.4) both in the AP and in the mortise view among all the examiners. Associating the two x-rays, the agreement remained poor. In the cases in which there was a diagnosis of articular penetration, there was a poor agreement related to which of the screws was intra-articular. The number of "inconclusive" responses was low and constant, without a statistically significant difference between the subspecialists (p > 0.05) [18].

## Discussion

Malleolar ankle fractures are extremely common injuries. The therapeutic decision in these cases is based primarily on simple and well-defined radiographic evaluations, which include AP, mortise, and lateral views. However, little has been said about intraoperative evaluation in these situations, either in relation to the quality of the reduction or in relation to the intra-articular penetration of the implants used to fix these fractures [19]. In addition, most of the comparable studies used retrospective models designs, reduced number of cases, lack of characterization in the evaluation of images, and the absence of statistical analysis.

Motta et al used three radiographic views on anteroposterior plane to retrospectively analyze the x-rays of 17 patients with the objective of evaluating the position of the screws used to fix the medial malleolar fractures of the ankle [13]. They observed that the AP view with the foot in a neutral position is better, since a real image of the medial clear space is obtained, facilitating the detection of articular penetration of the implants [13]. Gourineni et al compared the sensitivity of the anteroposterior views of the ankle with different rotations of the foot in the diagnosis of intra-articular penetration of the implants used to fix medial malleolar fractures [12]. These authors recommend the adoption of the AP view with neutral rotation in order to adequately evaluate the positioning of the implants used in the treatment of transversal medial malleolar fractures [12]. Romiti and Leitschuh performed nine different radiographic views of the ankle in order to study a human tibia removed from a skeleton with the objective of observing whether the synthesis material introduced into the medial malleolus was intra-articular [14]. When the hardware material was found positioned near the concave surface of the medial malleolus in any of the images, so it is extra-articular.

In the present experiment, we observed a low level of intra and interobserver agreement with relation to the diagnosis of articular penetration by the screws used to fix the medial malleolar fractures, both in terms of the AP and the mortise views. The agreement remained low when associating the two views. Furthermore, in the cases in which there was a diagnosis of articular penetration, there was a poor level of agreement in relation to which of the screws was intra-articular. When asked which radiographic view they were seeing, all examiners responded with good agreement. The number of "inconclusive" responses was low and constant during the two readings, showing that the drawing and the performance of the study were well done. Trauma surgeons showed a significantly lower number of "inconclusive" responses than Foot and Ankle Surgeons (p < 0.05), maybe reflecting the need of fast decisions of the prior specialists leading with trauma situations in the Emergency Departments.

There are some strengths in our study. In relation to the model used in this study, we followed the STARD Committee standards, which recommend the comparison of the results of one or more tests under evaluation with the results of the reference standard for diagnostically sensitive studies [15]. In the specific case of our investigation, the reference standard was the clinical exam, since we knew the location of the screws in each specimen.

We were unable to identify which intraoperative imaging is the best to determine whether screws are in or out of the joint. This can be seen as one weakness in our experiment. However this was not our aim and we feel it can be investigated in a future study.

## Conclusion

The rigorous evaluation of the two studied radiographic views allows us to suggest that they are not reliable for intraoperative diagnosis of the articular penetration of implants used to fix transverse medial malleolar fractures of the ankle. We recommend that one does not waste time performing radiographies on different rotations of the foot when invasion of the medial articular space is suspected. In these cases, the best to do in our opinion is to reposition the screw(s) in question.

Nevertheless, we advocate the routine use of all standard ankle views (AP, mortise, and lateral) during the surgical procedure, either with fluoroscopy or plain films. This is not our aim to change this investigation pattern, but to alert for the poor detection of articular penetration with these views.

## Competing interests

The authors declare that they have no competing interests.

## Authors' contributions

VG and AFSG designed the study and performed the experiments. VG, RPA and RESP evaluated the results and drafted the first version of the manuscript. NPA contributed to revisions of the manuscript. All authors read and approved the final version of the manuscript.
